# Vitamin D Deficiency or Supplementation and the Risk of Human Herpesvirus Infections or Reactivation: A Systematic Review and Meta-analysis

**DOI:** 10.1093/ofid/ofaa570

**Published:** 2020-12-22

**Authors:** Liang-Yu Lin, Ketaki Bhate, Harriet Forbes, Liam Smeeth, Charlotte Warren-Gash, Sinéad M Langan

**Affiliations:** Faculty of Epidemiology & Population Health, London School of Hygiene and Tropical Medicine, London, UK

**Keywords:** cytomegalovirus, Epstein, Barr virus, herpes zoster, herpesviridae, systematic review, vitamin D deficiency, vitamin D supplementation

## Abstract

**Background:**

Vitamin D may protect against respiratory virus infections, but any association with herpesviruses is unclear.

**Methods:**

We undertook a systematic review of vitamin D deficiency or supplementation and the risk of 8 human herpesviruses. Six databases and 4 gray literature databases were searched for relevant cohort studies, case–control studies, and clinical trials.

**Results:**

Ten studies were included, all conducted among immunosuppressed patients. There was no evidence that vitamin D deficiency is associated with cytomegalovirus (CMV) disease (pooled risk ratio, 1.06; 95% CI, 0.66–1.7), herpes zoster after transplantation (1 study), or HHV-8 among HIV patients (1 study). Vitamin D supplementation may decrease herpes zoster among hemodialysis patients (1 study) or CMV disease after renal transplantation (1 study), but supplementation was not associated with reduced EBV viral load among multiple sclerosis patients (1 study).

**Conclusions:**

Any association between vitamin D and herpesviruses remains inconclusive. Further studies in the general population are needed.

Herpesviruses are a family of 8 DNA viruses that induce lifelong latency after infecting humans; they include herpes simplex virus type 1 and type 2 (HSV-1 and HSV-2), varicella-zoster virus (VZV), Epstein-Barr virus (EBV), cytomegalovirus (CMV), HHV-6, HHV-7, and Kaposi’s sarcoma–associated herpesvirus (KSHV). These viruses can reactivate and lead to clinical symptoms when the immunity of the host declines [1]. Although many primary herpesvirus infections are mild or self-limited, both infection and reactivation may lead to rare but serious complications that affect quality of life and lead to a substantial burden on health care services. For example, VZV causes chickenpox among children and herpes zoster in adults. Especially among people older than age 65 years, zoster may lead to post-herpetic neuralgia, which is associated with an increased risk of cardiovascular outcomes and financial burden [2, 3]. CMV infection is usually asymptomatic in healthy adults, but infection of immunocompromised hosts can lead to graft loss or death [4]. Consequently, it is important to explore the immunomodulatory factors associated with infection or reactivation of herpesviruses.

Vitamin D is synthesized by the skin after sunlight exposure, or it can also be consumed through food or supplements. Its concentration in the blood is greatly affected by season and latitude as well as nutritional intake. This vitamin plays an essential role in absorbing calcium and phosphate, which are important to bone health. Vitamin D deficiency may lead to rickets in children or osteomalacia in adults [5]. Currently, no consensus exists about the threshold serum levels for defining vitamin D deficiency. Some studies have defined vitamin D deficiency as serum 25(OH)D levels <50 nmol/L^6^, while other studies and Public Health England recommendations have used 25(OH)D levels <25 nmol/L as their cutoff [7, 8]. To protect bone health, Public Health England recommends people taking 10 μg (400 IU) of vitamin D every day in the winter [9].

In addition to bone health, some studies have shown that this vitamin may have some immunomodulatory effects and anti-infective potential. At a cellular level, some studies have shown that vitamin D regulates the production of the antimicrobial peptide cathelicidin [10–13], and 1 study indicated that vitamin D supplementation was associated with a decrease in HSV-1 viral load and mRNA expression in HSV-1-infected cells [14]. In addition, among epidemiological studies, a meta-analysis showed that the risk of infection was lower in chronic kidney disease patients with normal or higher serum vitamin D levels [15], and another meta-analysis using original patient data from 25 randomized controlled trials showed that people receiving vitamin D supplementation had a lower risk of respiratory tract infection [16].

However, whether vitamin D is associated with protection against herpesviruses is still unclear, and no comprehensive review exists of this possible association. As vitamin D supplementation is an inexpensive public health intervention, studying its possible association with herpesviruses may help us find a novel approach to mitigate the health impact of these infectious diseases. We therefore undertook a systematic review to examine the relationship between serum vitamin D levels or oral vitamin D supplementation and the risk of infection with or reactivation of any of the 8 human herpesviruses.

## METHODS

### Protocol and Registration

The protocol of this study has been previously registered on PROSPERO (registration number: CRD42019130153) and published [17].

### Eligibility Criteria

As previously described, we included only human studies in our review [17]. The exposures were serum vitamin D deficiency or oral vitamin D supplementation, including vitamin D analog treatment. Vitamin D deficiency was defined as serum 25(OH)D <25 nmol/L, to be consistent with the Public Health England approach. The comparator for vitamin D–deficient participants was people with sufficient serum vitamin D levels, and the comparator for vitamin D supplementation was participants without vitamin D supplementation or a placebo group. The outcomes were infection or reactivation of any human herpesvirus, confirmed based on physicians’ clinical diagnoses or by laboratory-based techniques such as polymerase chain reaction. For more rigorous causal estimation, only cohort, case–control, and intervention studies were eligible to be included.

### Information Sources and Search

One researcher (L.Y.L.) searched 6 main databases and 4 gray literature databases. The main databases were Medline (Ovid), EMBASE (Ovid), Web of Science, Scopus, the Cochrane Library, and Global Health (Ovid). The gray literature databases included Open Grey, BASE, EThOS, and the clinical trials register at ClinicalTrials.gov. The search was updated to August 31, 2019.

The search strategy was reviewed and revised by a librarian. Synonyms of “human herpesviruses” and “vitamin D” were searched using both controlled vocabularies and keywords in different databases, in which subject headings were modified. The search results were combined using the Boolean logic operator “AND.” One author (L.Y.L.) also hand-searched the final included studies to identify potential eligible articles. Duplicated search results were removed using EndNote X9 [18].

### Study Selection, Data Collection, and Data Items

The titles and abstracts of the included articles were independently screened by 2 researchers (L.Y.L. and K.B.), and the full texts of the eligible studies were further examined. Any discrepancy in these processes was resolved by a third researcher (S.M.L.). Two researchers (L.Y.L. and K.B.) independently extracted data from the first 3 studies using a predefined form, and 1 researcher (L.Y.L.) extracted the other included articles. The complete data extraction form is available in the Supplementary Data. Briefly, data were extracted following the framework of population, exposure/intervention, comparator, and outcomes. In addition, study characteristics such as study design, study population, results, statistical analysis methods, and confounders were extracted. The numbers of subjects with outcomes were obtained for each exposure group, and the reported crude results and adjusted results were recorded. If a study reported >1 vitamin D outcome, such as deficiency, insufficiency, and sufficiency, only the outcomes of vitamin D deficiency and sufficiency were extracted. For studies reporting continuous outcomes, their mean or median change of outcome and standard deviation were recorded. The authors of the included studies were contacted for unclear or missing data.

### Risk of Bias in Individual Studies

Predefined templates based on the Cochrane approach were used to assess the risk of bias in the included studies. For observational studies, 5 domains were assessed: confounding factors, selection of participants, misclassification of variables, bias due to missing data, and reverse causation. For intervention studies, bias was assessed using version 2 of the Cochrane risk-of-bias tool for randomized trials (RoB 2) to assess random sequence generation; allocation concealment; blinding of participants, personnel, and assessment; incomplete outcome data; and selective reporting [19]. The assessment tools were piloted and tested, and the risk of bias of the first 3 included studies was assessed by 2 independent researchers (L.Y.L. and K.B.) to ensure consistency and quality. One researcher (L.Y.L.) evaluated the risk of bias of the other studies. Discrepancies in the assessment were resolved by a third researcher (S.M.L. or C.W.G.).

### Summary Measures

The results of the included studies were synthesized by vitamin D exposure and by different herpesviruses using a narrative approach. The main adjusted risk ratio (RR), hazard ratio (HR), or odds ratio (OR) was presented for each study. If the numbers of outcomes among exposure and comparator groups were reported without an estimate of relative risk, the unadjusted risk ratio and 95% confidence interval were calculated. For the studies with similar study designs, exposures, and outcomes, we further assessed their heterogeneity and synthesized them using a meta-analysis. The heterogeneity between studies was assessed using *I*^2^, and a random-effects meta-analysis was used for substantial heterogeneity (*I*^2^ > 50%). A funnel plot was used to evaluate publication bias [20]. All analysis was conducted using Stata, version 16/IC (StataCorp, College Station, TX, USA).

### Risk of Bias Across Studies

For studies reporting similar exposures and outcomes, we further analyzed the risk of bias. The Grading of Recommendations, Assessment, Development and Evaluations (GRADE) framework was used to assess the overall quality of evidence [21]. The levels of confidence were established through the design of included studies, which were further downgraded or upgraded according to the domains of risk of bias, inconsistency, indirectness, imprecision, publication bias, strong association, dose response, and confounding. A rank of “high,” “moderate,” “low,” or “very low” was given to the quality of evidence.

### Patient Consent Statement

Patient consent is not required for publication. Ethical review is not required for a systematic review.

## RESULTS

### Study Characteristics

**Figure 1.**
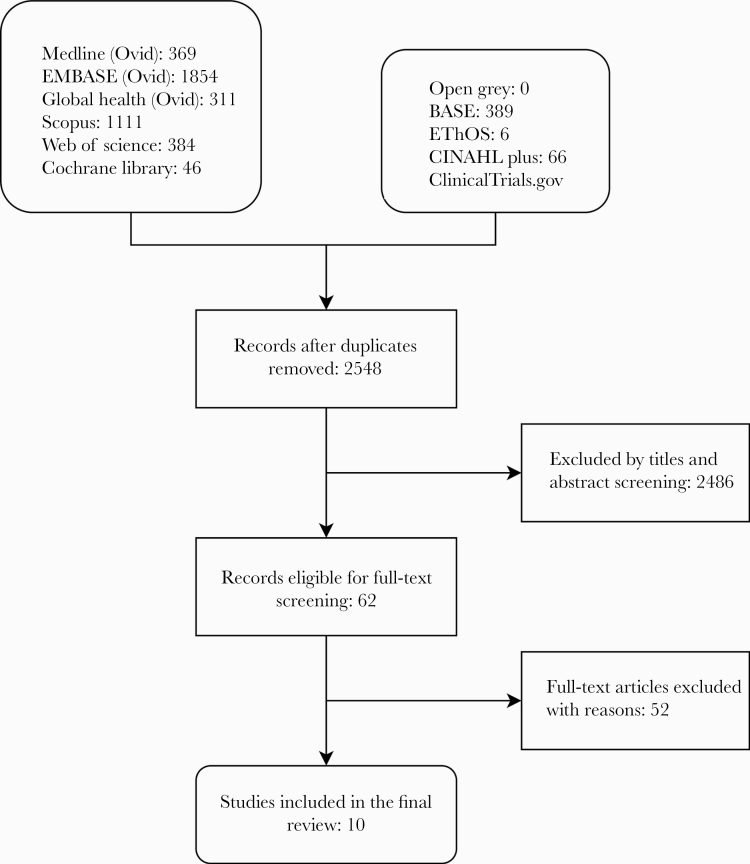
Flow diagram of study selection.

Figure 1 displays the steps to study selection. In our search, 4537 articles were initially identified. After removing duplicated studies, we scanned 2548 titles and abstracts. We excluded articles that did not meet our inclusion and exclusion criteria, and we reviewed the full text of 62 articles for eligibility.

Ten studies were finally included in our review, and their characteristics are summarized in Table 1. They were 8 cohort studies, 1 case–control study, and 1 randomized controlled trial. All cohort and case–control studies were undertaken in single-hospital settings, while the only randomized controlled trial used data from 5 hospitals in the Netherlands. All studies were conducted in different regions: 4 studies from Asia, 3 from Europe, 2 from North America, and 1 from Africa. All studies were conducted with patients with the following underlying health conditions: end-stage renal disease requiring hemodialysis (n = 1), organ transplants (n = 7), multiple sclerosis (n = 1), and HIV (n = 1). Seven included studies analyzed the association between serum vitamin D levels and herpesvirus infections, and the other 3 studies assessed the correlation between vitamin D supplementation and herpesviruses.

**Table 1.** Characteristics of the Included Studies

Author, Year	Design	Study Period	Setting	Study Population at Recruitment	Intervention/Exposure Definition and Ascertainment	Comparator Definition and Ascertainment	Outcome Type	Outcome Definition and Ascertainment
Chao et al. (2012) [24]	Case–control study	January 2000 to December 2009	Taiwan, single-hospital study	Patients received dialysis	Vitamin D supplementation (1α-hydroxylated vitamin D)	No vitamin D supplementation	Primary	Clinically diagnosed herpes zoster
Erlandson et al. (2014) [23]	Historical cohort	June 2003 to May 2005	Zimbabwe, single-hospital study	Adult patients with HIV-1 and Kaposi’s sarcoma	Serum 25(OH)D levels (nmol/L):	Serum vitamin D levels (nmol/L):	Secondary	HHV-8 viral load in plasma and peripheral blood mononuclear cells
					• Deficiency: ≤50	• Adequate ≥75 nmol/L		
Lee et al. (2014) [43]	Historical cohort	January 2005 to December 2010	USA, single-hospital study	Patients received kidney transplantation	Serum 25(OH)D levels (ng/mL):	Serum 25(OH)D levels (ng/mL):	Secondary	Laboratory-confirmed CMV disease
					≤ 20	> 20		
Saber et al. (2015) [33]	Prospective cohort study	June 2013 to December 2013	Iran, single-hospital study	Potential kidney transplant patients	Serum 25(OH)D levels (ng/mL):	Serum 25(OH)D levels (ng/mL):	Primary	Laboratory-confirmed CMV infection
					• Deficiency: <15	• Normal: >30		
Moscarelli et al. (2016) [25]	Historical cohort	May 2005 to August 2014	Italy, single-hospital study	Patients received kidney transplantation	Vitamin D supplementation (calcitriol)	No vitamin D supplementation	Primary	Laboratory-confirmed CMV infection
Ban et al. (2017) [22]	Historical cohort	January 2011 to December 2013	Korea, single-hospital study	Patients received kidney transplantation	Serum 25(OH)D levels in low tertile groups (≤8.3 ng/mL)	25(OH)D levels in the high tertile group (>12.1 ng/mL)	Secondary	Laboratory-confirmed CMV infection and clinically diagnosed herpes zoster
Park et al. (2017) [32]	Historical cohort	January 2011 and December 2013	Korea, single-hospital study	Patients received kidney transplantation	Serum 25(OH)D levels (ng/mL):	Serum 25(OH)D levels (ng/mL):	Primary	Laboratory-confirmed CMV infection
					<20	≥20		
Rolf et al. (2018) [26]	Randomized controlled trial	March 2011 to February 2014	The Netherlands, a substudy of a clinical trial	Patients with relapsing-remitting multiple sclerosis	Vitamin D supplementation (calcitriol)	Placebo	Secondary	EBV viral loads in peripheral blood mononuclear cells and B cells
Astor et al. (2019) [35]	Historical cohort	January 2004 to June 2014	USA, single-hospital study	Adult patients (age >18 y) received kidney transplantation	Serum 25(OH)D levels (ng/mL):	Serum 25(OH)D levels (ng/mL): ≥30	Primary	Laboratory-confirmed CMV infection
					Deficiency: <20			
Fernandez-Ruiz et al. (2019) [34]	Prospective cohort	November 2014 to December 2016	Spain, single-hospital study	Patients received kidney transplantation	Serum 25(OH)D levels (ng/mL): <20	Serum 25(OH)D levels (ng/mL): ≥20	Primary	Laboratory-confirmed CMV disease

Abbreviations: CMV, Cytomegalovirus, HIV, human immunodeficiency virus.

^a^1 nmol/L = 0.4 ng/mL.

Among studies assessing serum vitamin D status and the risk of different herpesvirus infections or reactivation, the definition of vitamin D deficiency varied. Five cohort studies defined vitamin D deficiency as serum 25(OH)D below 20 ng/mL or 50 nmol/L, 1 study used vitamin D levels <15 ng/mL (37.5 nmol/L), and the other used tertiles to classify its participants, with the mean vitamin D level in the lowest tertile being 20.78 nmol/L.

Among studies of vitamin D supplementation and the risk of herpesviruses infection or reactivation, 2 used activated vitamin D analog (calcitriol or 1α-hydroxylated vitamin D) supplementation, and the other used inactive vitamin D3 (cholecalciferol) supplementation (Table 1).

### Meta-analysis of Vitamin D Deficiency and the Risk of CMV Infection or Reactivation Among Patients With Organ Transplants

**Figure 2.**
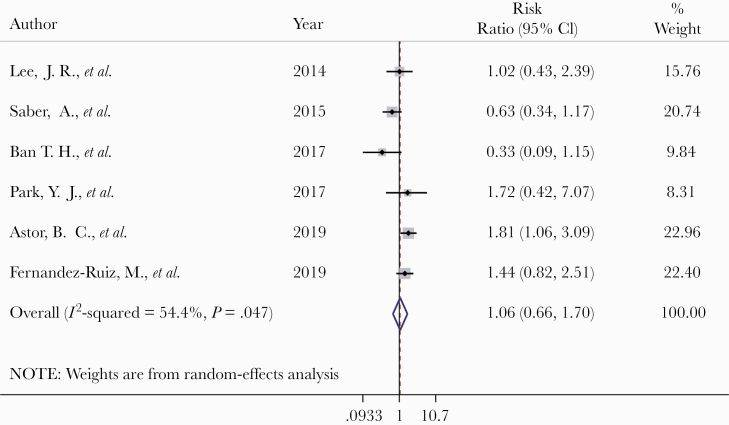
Forest plot of the summary of the effects of vitamin D deficiency on CMV risk after transplantation. Abbreviations: CMV, cytomegalovirus; RR, risk ratio.

**Figure 3.**
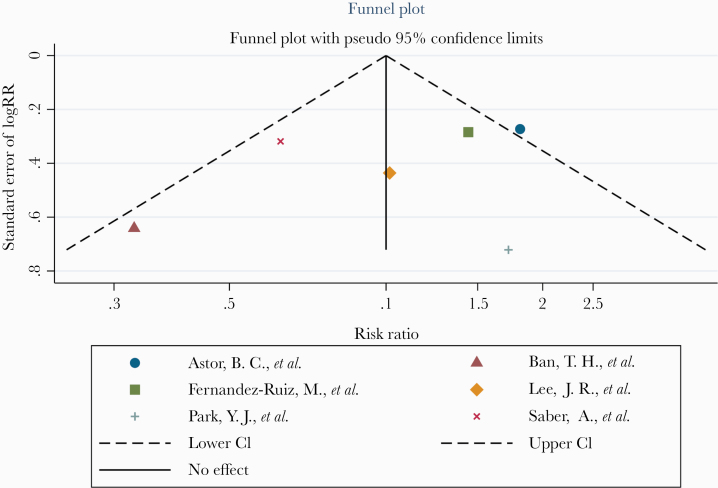
Assessment of publication bias of serum vitamin D levels before transplantation and the risk of CMV infection after transplantation. Abbreviation: CMV, cytomegalovirus.

A random-effects meta-analysis was conducted to pool 6 observational studies, which analyzed the association between serum vitamin D deficiency and the risk of CMV disease after transplantation therapy. No evidence was found that serum vitamin D deficiency increased the risk of CMV infection or reactivation in patients receiving transplants (RR, 1.06; 95% CI, 0.66–1.70) (Figure 2). The sample sizes of these studies were relatively small (Table 2), and the heterogeneity between studies was high (*I*^2^ = 55.4%). Five of the included studies did not adjust for confounding factors, and bias due to missing data was unclear in 2 studies (Table 3). Due to the high risk of bias and imprecise estimates of these studies, the overall quality of evidence was low (Table 4). The funnel plot of the included studies showed a relatively symmetric pattern, which suggests that the risk of publication bias was low (Figure 3).

**Table 2.** Summary of Results

Author, Year	Population Size, No.; Mean/Median Follow-up Time, mo	Subjects With Outcome [or Exposure for Case–Control Studies], No. (%)	Statistical Analysis Method Used	Main Reported Results	Covariates Adjusted for:
Vitamin D deficiency					
Serum vitamin D levels before transplantation and the risk of CMV infection after transplantation					
Lee et al. (2014)	n = 351; followed for 12 mo	Deficiency: n = 13/216 (6%)	Not reported	Risk ratio, 1.02 (95% CI, 0.43–2.39) [calculated by review authors]	Not adjusted
		Sufficiency: n = 8/135 (5.9%)			
Saber et al. (2015)	n = 82; followed for 4 mo	Deficiency: n = 15/41 (37%)	Not reported	Risk ratio, 0.6 (95% CI, 0.37–0.96) [calculated by review authors]	Not adjusted
		Sufficient: n = 7/12 (58%)			
Ban et al. (2017)	n = 174; median follow-up period 35.5 mo	CMV infection: Low tertile: n = 3/59 (5.1%)	Not reported	CMV:	Not adjusted
		High tertile: n = 9/58 (15.8%)		Risk ratio, 0.328 (95% CI, 0.09–1.15) [calculated by review authors]	
Park et al. (2017)	n = 164; followed for 24.8 mo	Deficiency: n = 16/135 (11.9%)	Not reported	CMV:	Not adjusted
		Sufficiency: n = 2/29 (6.9%)		Risk ratio, 1.72 (95% CI, 0.42–7.07) [calculated by review authors]	
Astor et al. (2019)	n = 1976; followed for 12 mo	Not reported	Cox proportional hazard regression models	Serum 25(OH)D ≥30 ng/mL: reference	Age, sex, ethnicity, cause of ESKD, BMI, donor status, prior transplant, delayed graft function, induction immunosuppression, smoking status, HLA mismatch category, CMV serostatus, time from transplant to 25(OH)D measurement, history of acute rejection, estimated glomerular filtration rate category, season, maintenance immunosuppression, and quartile of calcineurin inhibitor level
				Serum 25(OH)D <20 ng/mL: 1.81 (95% CI, 1.06–3.09)	
Fernandez-Ruiz et al. (2019)	n = 215; followed up for at least 12 mo	Vitamin D deficiency: n = 34/135 (25.2%)	Not reported	CMV:	Not adjusted
		No deficiency: 14/80 (17.5%)		Risk ratio, 1.44 (95% CI, 0.82–2.51) [calculated by review authors]	
Serum vitamin D levels before transplantation and the risk of herpes zoster after transplantation					
Ban et al. (2017)	n = 174; median follow-up period 35.5 mo	Herpes zoster:	Not reported	Herpes zoster:	Not adjusted
		Low tertile: n = 6/59 (10.2%)		Risk ratio, 0.98 (95% CI, 0.337–2.87) [calculated by review authors]	
		High tertile: n = 6/58 (10.3%)			
Serum vitamin D levels and the change of serum HHV-8 viral load					
Erlandson et al. (2014)	n = 85; followed for 24 mo	Not applicable	Mann-Whitney test	A decrease in HHV-8 viral load (log), median (IQR): In plasma:	Not adjusted
				• Inadequate: 0.5 (0–1.5)	
				• Adequate: 0.4 (0–1.5)	
				In PBMC:	
				• Inadequate: 0.4 (0–1.5)	
				• Adequate: 1.0 (0–2.0)	
Vitamin D supplementation					
Vitamin D supplementation and the risk of herpes zoster among dialysis patients					
Chao et al. (2012)	n = 126; followed at least 1 mo before the event	Exposure among HZ cases: 3/63 (5.4%); exposure among controls: 29/63 (46%)	Conditional logistic regression model	OR, 0.06 (95% CI, 0.0–0.4)	Hepatitis or cirrhosis, cerebrovascular accident, use of iron therapy, use of corticosteroids, use of statins, CRP, intact PTH, ferritin
Vitamin D supplementation and risk of CMV infection after transplantation					
Moscarelli et al. (2016)	n = 360; followed for 12 mo	Nonuser group: n = 21 (9%); user group: n = 4 (3%)	Cox proportional hazards regression	HR, 2.31 (95% CI, 1.44–3.71)	Serum 1,25(OH)_2_D3 deficiency, biopsy-proven acute rejection, BKV infection, CMV serostatus, steroid boluses, BMI
Vitamin D supplementation and change of EBV viral load in blood cells					
Rolf et al. (2018)	n = 53; followed for 7.3 mo	Not applicable	Mann-Whitney *U* test	Fold change relative to T0:	Not adjusted
				Treatment: 1.38 (0.36–3.11)	
				Placebo: 1.31 (0.16–3.17)	

Abbreviations: CMV, Cytomegalovirus; CI, confidence interval; HR, hazard ratio; HHV-8, Human herpesvirus-8; OR, odds ratio; PBMC, peripheral blood mononuclear cell.

^a^1 nmol/L = 0.4 ng/mL.

**Table 3.** Assessment of Bias for Individual Studies

Observational Studies						
Included Studies		Confounding	Selection of Participants	Misclassification of Variables	Bias due to Missing Data	Reverse Causation
Serum vitamin D levels before transplantation and the risk of CMV infection after transplantation						
Lee et al. (2014)		High	Low	Low	Low	Low
Saber et al. (2015)		High	High	Low	Unclear	Low
Ban et al. (2017)		High	Low	Moderate	Low	Low
Park et al. (2017)		High	Low	Low	Low	Low
Astor et al. (2019)		Low	Low	Low	Low	Low
Fernandez-Ruiz et al. (2019)		High	Low	Low	Unclear	Low
Serum vitamin D levels and the risk of herpes zoster among transplantation patients						
Ban et al. (2017)		High	Low	Moderate	Low	Low
Serum vitamin D levels and the change of serum HHV-8 viral load						
Erlandson et al. (2014)		High	High	Low	Moderate	Low
Vitamin D supplementation and the risk of CMV infection after transplantation						
Moscarelli et al. (2016)		Low	Low	Moderate	Unclear	Low
Vitamin D supplementation and the risk of herpes zoster among dialysis patients						
Chao et al. (2012)		Moderate	Low	Low	Low	Low
Interventional study						
Included Studies	Randomization Process	Deviations From Intended Interventions	Missing Outcome Data	Measurement of the Outcome	Selection of the Reported Result	Overall Judgment
Vitamin D supplementation and change of EBV viral load in blood cells						
Rolf et al. (2018)	Some concerns	High	High	Some concerns	Some concerns	High

Abbreviations: CMV, Cytomegalovirus; EBV, Epstein-Barr virus; HHV-8, Human herpesvirus-8.

**Table 4.** Quality of Evidence of Outcomes

No. of Studies	Study Design	Risk of Bias	Inconsistency	Indirectness	Imprecision	Publication Bias	Other Considerations	Quality
Outcomes: vitamin D deficiency/insufficiency associates with an increased risk of CMV infection								
6	Observational study	Serious	Serious	Not serious	Serious	Not serious	None	Very low

Abbreviation: CMV, Cytomegalovirus.

^a^Heterogeneity is substantial (*I*^2^ = 55.4%; *P* = .047).

### Vitamin D Deficiency and Herpesvirus Infection or Reactivation

One cohort study [22] also investigated whether vitamin D deficiency was associated with the risk of developing herpes zoster after kidney transplantation, showing no evidence of association (RR, 0.98; 95% CI, 0.337–2.87). Another study investigated the association between serum vitamin D levels and change in HHV-8 viral load among HIV-positive patients recruited from a trial [23]. It showed no evidence that inadequate vitamin D levels were associated with higher viral load in plasma (viral load decreased by 0.5 [0 to 1.5] log copies/mL in the inadequate vitamin D group and by 0.4 [0 to 1.5] log copies/mL in the adequate vitamin D group; *P* = .8) or in peripheral blood mononuclear cells (viral load decreased by 0.9 [–0.4 to 2.3] log copies/mL among the inadequate vitamin D group and by 1.0 [0 to 2.0] log copies/mL in the adequate vitamin D group; *P* = .9) (Table 2). Nevertheless, both studies had a high risk of bias in at least 1 domain (Table 3).

### Vitamin D Supplementation and the Risk of Herpesvirus Infection or Reactivation

One case–control study (n = 126) indicated that hemodialysis patients receiving vitamin D supplementation had a lower risk of developing herpes zoster (OR, 0.06; 95% CI, 0.0–0.4) (Table 2) [24]. The assessed risk of bias in this study was moderate in 1 domain (Table 3).

A historical cohort study assessed the association between vitamin D supplementation before transplantation and the risk of CMV after transplantation [25]. The group unexposed to vitamin D supplementation had a 2.3-fold increased hazard of developing CMV disease after transplantation (HR, 2.31; 95% CI, 1.44–3.71) (Table 2). However, the risk of misclassification in this study was moderate, and the risk of bias due to missing data was unclear (Table 3).

One randomized controlled trial assessed the effect of oral vitamin D supplementation on EBV viral load in blood [26], showing that high-dose vitamin D supplementation did not decrease the viral load in peripheral blood mononuclear cells or B cells (Table 2). However, the overall risk of bias of this interventional study was high (Table 3).

## DISCUSSION

We reviewed 10 studies examining serum vitamin D deficiency or vitamin D supplementation and the risk of herpesvirus infection or reactivation among patients with comorbidities. The results showed no consistent association between serum vitamin D deficiency and the risk of herpesvirus infection or reactivation, but some evidence that vitamin D supplementation may be associated with a reduced risk of herpes zoster or CMV disease. However, the risk of bias of most included studies was high. Therefore, the evidence for establishing an association between vitamin D deficiency or oral vitamin D supplementation and the risk of herpesvirus infection or reactivation is still inconclusive.

Ours is the first systematic review of vitamin D and herpesvirus infection or reactivation. Some previous studies have also explored the various association between vitamin D deficiency or vitamin D supplementation and similar chronic viral infections. Among patients with chronic hepatitis B virus infection, a meta-analysis showed that serum vitamin D levels were negatively associated with hepatitis B viral load [27]. For hepatitis C virus patients, 1 meta-analysis reported that baseline serum vitamin D was associated with a sustained virologic response to antiviral and interferon treatment [28], while another recent meta-analysis reported no association [29]. A previous systematic review found no clear evidence of an effect of vitamin D supplementation on HIV viral load [30]. The immunomodulatory mechanism of vitamin D is still unclear, and its potential for preventing clinical viral infections is also inconclusive. Due to the paucity of current literature, further research is needed.

Our meta-analysis found no evidence that vitamin D status affected the risk of CMV infection or reactivation among patients receiving transplantation. CMV and other herpesviruses are the most common viral infections among transplant recipients [31]. However, the detected incidence of CMV will vary according to the use of prophylactic measures, differences in testing frequency, and definitions of CMV disease. These factors may have contributed to heterogeneity in our analysis. There were 2 studies that did not mention prophylactic measures [32, 33], and 1 study provided prophylactic antiviral treatment only for people with a high risk of CMV disease [34]. Two studies regularly checked participants’ CMV viral loads [22, 25], and 3 studies only examined CMV antigen or viral load when patients were symptomatic [33–35]. These inconsistencies may lead to different estimations of the risk of CMV infections. We suggest that future studies about vitamin D and CMV diseases consider using a standardized definition of CMV and a consistent follow-up approach [36], so that researchers can accurately estimate the association between vitamin D and CMV infections among transplant patients.

While our study did not focus on the outcome of transplant rejection, there is a close relationship between CMV infection and graft injuries, which increase the risk of rejection [37, 38]. A recent systematic review also indicated that there was weak evidence showing an association between vitamin D deficiency and acute or chronic graft-vs-host disease (GVHD) [39]. While viral infections such as CMV may act as mediators of any relationship between vitamin D and graft loss, further research into these complex relationships is needed.

The effects of vitamin D supplementation on the risk of EBV remain inconclusive. The only included study did not find a reduction in EBV viral load among multiple sclerosis patients randomized to receive high-dose vitamin D supplementation, but levels of antibody against EBV were reduced in this intervention group [26]. While this may provide some limited evidence for an immunomodulatory effect of vitamin D, it is unclear how well EBV antibodies reflect infection status, so further studies are needed.

Because of the impairment of renal function in calcium and phosphate homeostasis, many CKD patients take vitamin D supplements for maintaining bone health [40]. One included study showed that taking vitamin D supplements reduced the risk of herpes zoster among CKD patients receiving hemodialysis [24]. In this study, all participants were recruited before the introduction of zoster vaccine in Taiwan [41]. However, as zoster vaccinations can effectively reduce the risk of herpes zoster among older patients with CKD [42], future studies of the relationship between vitamin D and herpes zoster should take zoster vaccinations into account.

The studies included in our review have some limitations. First, 7 observational studies did not adjust for possible confounding factors, and they did not report herpesvirus infections as their main outcomes. In addition, the only included trial did not report the details of randomization or show a clear baseline characteristics table, so it was not possible to assess the effectiveness of the randomization process. These unadjusted confounding factors may lead to a high risk of bias in assessing the association. According to the included studies that adjusted for potential confounders, some evidence existed that vitamin D supplementation may be associated with a decreased risk of CMV disease among transplantation patients and herpes zoster among dialysis patients [24, 25]. In addition, among patients who received organ transplants, vitamin D deficiency was associated with a higher hazard of CMV disease after adjusting for confounders [35].

The inconsistent definition of vitamin D deficiency is another major limitation. Of 7 observational studies assessing vitamin D deficiency, 5 defined vitamin D deficiency as serum 25(OH)D <50 nmol/L (20 ng/mL), 1 defined it as 15 ng/mL (37.5 nmol/L), and the other used the lowest tertile as the exposure group, which was serum 25(OH)D <8.3 ng/mL (20.75 nmol/L). This significant difference in exposure definitions increases the heterogeneity between studies.

Another limitation is generalizability. The populations of the included studies were people with severe underlying conditions, such as end-stage renal disease, organ transplantation, and HIV. Further, these studies were conducted in single-hospital settings. Their results cannot be extrapolated to other populations with different comorbidities or even the general population. More studies among different populations are needed.

Our review has some strengths. This is the first review systemically examining the existing available evidence about vitamin D and herpesvirus infection. We comprehensively searched 6 major medical databases and 4 gray literature databases, and we summarized the results and assessed the risk of bias using a predefined framework. However, our study has some limitations. First, due to the paucity of studies, we were not able to review the association between vitamin D and some herpesvirus infections, such as HSV-1, HSV-2, HHV-6A, HHV-6B, and HHV-7. This may be caused by their difficulty in diagnosis. Second, because no guideline exists for defining serum 1,25-dihydroxycholecalciferol, an active metabolite of vitamin D, we were unable to include studies assessing this. Third, despite the comprehensiveness of our search, our search strategy may still have missed some eligible studies. Although we did not limit the language for eligible studies, studies in other languages may not be able to be identified. Further reviews of the association between vitamin D and herpesviruses need to consider these limitations.

Based on currently available studies, some limited evidence suggests that vitamin D supplementation may have a protective effect against herpes zoster in hemodialysis patients and CMV disease in renal transplant patients, but insufficient evidence supports any association between serum vitamin D deficiency and the risk of herpesvirus infection or reactivation. However, the current studies have focused solely on immunosuppressed patients with major underlying comorbidities, and some did not adjust for potential confounding factors. As vitamin D deficiency is not uncommon, for future studies, it is important to focus not only on individuals with specific comorbidities but also on the general population. In addition, future studies need to adopt consistent definitions of vitamin D deficiency, and adequately adjust for possible confounding factors, to provide robust evidence of any association between vitamin D and herpesvirus infection or reactivation.

## Supplementary Data

Supplementary materials are available at *Open Forum Infectious Diseases* online. Consisting of data provided by the authors to benefit the reader, the posted materials are not copyedited and are the sole responsibility of the authors, so questions or comments should be addressed to the corresponding author.

ofaa570_suppl_Supplementary-Tables
