# Supplementary material for: Vitamin D Deficiency or Supplementation and the Risk of Human Herpesvirus Infections or Reactivation: A Systematic Review and Meta-analysis
Source: Open Forum Infect Dis. 2020 Dec 22;8(1):ofaa570. doi: 10.1093/ofid/ofaa570 (PMC7817081; doi:10.1093/ofid/ofaa570)
Supplement: ofaa570_suppl_Supplementary-Tables [file ofaa570_suppl_supplementary-tables.pdf]

Author, Year	Sponsorship	Design	Aims and Objectives	Study period, and length of follow-up time	Setting	Study population at recruitment and sampling methods	General study population characteristics	Inclusion criteria (study population at recruitment)	Exclusion criteria (study population at recruitment)	Exposure Definition and ascertainment	Comparators Definition and ascertainment	Outcome Type	Outcome definition and ascertainment	Other	Population size (N), follow-up time (years)	Enrolled study population characteristics: AGE	Enrolled study population characteristics: sex	Enrolled study population characteristics	Subjects with the outcome (N, %), or exposure for case-control studies	For continuous outcome: mean/median/percentage (SD) change	Lost to follow up or withdrawing from trials	The statistical analysis method used	Main reported crude results (RR, OR, HR and CI)	Main reported adjusted results	Is dose-response seen	Confounders/covariates measured	Confounders/covariates adjusted for	Other additional stratified analysis/ subgroup analysis	Comments or notes
Erlandson, K. M., et al. (2014)	Hartford Foundation Center of Excellence, NIH, GSK	Historical cohort	To establish if a high prevalence of vitamin D deficiency is found in Zimbabweans with AIDS-KS and if low baseline Vit D would correlate with progression of AIDS-KS	June 2003 to May 2005. 96 weeks	Parirenyatwa Hospital PS Clinic in Harare, Zimbabwe (single hospital)	NI	NI	1) KS confirmed on histology 2) age>18 3) confirmed HIV-1 antibody 4) Hb >7.5g/dl 5)absolute neutrophil count >750 cells/micro litre, 6) agreement not ot participate in a conception process 7)ART na?ve 8) no chemotherapy 9) no Radiotherapy for less than or equal to 45 days before study entry 10) no intention to relocate	NI	Inadequate 25(OH)D: <75 nmol/l; adequate 25(OH)D: >=75 nmol/l	vitamin D Inadequate: <75 nmol/l	Secondary	HHV-8 plasma viral load (log) HHV-8 PBMC viral load (secondary outcomes)		85 Patients with baseline samples available; followed for 4 years (96 weeks)	Inadequate 25(OH)D 38 (33-45) n=45. Adequate 36 (31-43) n= 40	Inadequate 14 female 31 male, Adequate 12 female 30 male	BMI Inadequate 21.5 (20-22.8), adequate 21.5 (19-24.3)	90 (45 inadequate and 40 adequate)	*Decrease in HHV-8 plasma viral load (log) (median, IQR): inadequate: 0.5 (0-1.5) adequate: 0.4 (0-1.5) p=0.8 *Decrease in HHV-8 PBMC viral load (log)(median): inadequate: 0.4 (0-1.5) adequate: 1.0 (0-2.0) p=0.9	4 lost to follow up before week 24, 1 lost between 24 and 48 weeks and 6 lost between 48 and 96 weeks.	OR and 95% CI tested with a Chi suared or Fishers exact. Logisitic regression for confounders.	Inadequate 25(OH)D Dec in HHV-8 plasma viral load 0.5 (1-1.5). Decrease in HHV-8 PBMC viral load log 0.9 (-).4-2.3) P value HHV plasma 0.8, P-value HHV8 PBMC 0.9	Inadequate plasma viral load 0.5 (0-1.5) adequate 0.4 (0-1.5) PBMC viral load 0.9 (-0.4-2.3) adequate 1.0 (0-2.0)	No	HIV1 RNA and CD4 lymphocytes (for different analysis) Age gender BMI though does not say adjusted for	NI		
Rolf, L., et al. (2018)	Merck and Nationaal MS Fonds Nederland	RCT	To investigate the effect of high-dose vitamin D3 supplements on antibody levels against the EBV nuclear antigen-1 (EBNA-1) in patients with relapsing-remitting multiple sclerosis (RRMS) and to explore any underlying mechanism affecting	48 weeks	Netherlands for SOLARIUM but SOLAR Europe and 53 sites. Presume analysis for SOLARIUM was done in the Netherlands and just used patients recruited there	PRMS Patients for the SOLARIUM substudy were recruited in four of the five participating centers in the Netherlands	Patients with replapsing-remitting multiple sclerosis (PRMS) 18-55, treated with interferon - B1a, with first clinical evetn in previous 5 years and active disease, but no relapse 30 days before inclusion.	aged 18?V50 years; have a diagnosis of RRMS; have a brain and/or spinal MRIwith findings typical of MS; have a first clinical event occurringwithin 5 years prior to screening; have had at least relapse, or one or more Gd-enhancing or new T2 MRI lesions within the 12months prior to screening; have an EDSS score ?4.0 at screening; are currently and for the first	Pregnancy or lactation; any disease other than MS that could better explain signs and symptoms; complete transverse myelitis or bilateral optic neuritis; a relapse within 30 days prior to study day 1 (SD1); use of corticosteroids or adrenocorticotrophic hormone within 30 days prior to Study Day 1; abnormalities of vitamin D-	7000 iU/day in first 4 weeks followed by 14,000 iU/day up to week 48	Placebo	Secondary	Laboratory-confirmed (Quantification of EBV viral loads with qPCR)		n=53, 7.3 months (4.4-12)	37.5 (+/- 8.2)	F 35 M 18	Disease duration months 7.3 placebo 7.3 Vit D3. BMI > or equal to 25 13/23 placebo 16/30 Vit D3. EDSS week 0 comparison between < or equal to 3.5 Placebo 22 Vit D3 28, 4.0-5.5 Placebo 1 VItD3 2. Also see number attacks during last 2 years at week 0 and	RRMS Placebo 23, Vit D3 30, EBV load, placebo 19, Vit D3 24	Fold change relative to T0 (2??G?GCT) : placebo: 1.31 (0.16?V3.17) Treatment: 1.38 (0.36?V3.11)	2 placebo. 3 Vit D 3 group.	Group differences Mann-Whitney U test and within group differences between week 0 and week 48 Wilcoxon signed-rank test	NA	NA	No	Sex, age, disease duration, BMI, EDSS, attacks in last 2 years and duration since last attack, Caucasian origin (yes/no).	NA	NA	Table 2 showed the change of EBV viral load (continuous outcomes)

Saber, A., et al. (2015).	Nephrology and urology research center of Baqiatallah University	Prospective cohort study	To compare the level of vitamin D in two groups of patients with and without CMV infection within four months after transplantation. To find a relationship between vitamin D level, before and after transplantation in each group.	June 2013 - Dec 2013	Baqiyatallah hospital Tehran, Iran (a single hospital study)	Potential kidney transplant patients on immunosuppressive medication with and without CMV	Patients received renal transplantation; case: age 51; control: 44	Triple therapy with glucocorticoids, cyclosporin and mycophenolate mofetil. In some cases tacrolimus or azathiprine were used instead of cyclosporin and rapamycin instad of MMF.	Patients not compliance to refer, not taking drugs and irregular use of medications	Serum vitamin D levels: insufficiency: 15-30 ng/mL; deficiency:< 15ng/mL	Sufficiency: serum vitamin D over 30ng/mL	Primary	Laboratory defined: serum CMV Ag + or PCR	It's a cohort with multiple exposure measurement; they reported it like a case-control study	N=82; followed for 4 months	CMV infected 51 =/-14. CMV non infected 44 +/- 14	CMV infected 24 male 16 female. CMV non-infected 21 male and 21 female	received renal transplantation; casue of kidney failure includes: DM, HTN, PKD, nephrotic syndrome, renal stones, pyelonephritis, unknown	Deficiency: 15, 38% Insufficiency: 18, 45% Sufficient: 7, 17%		NI	Chi-square	NA	NA	No	Age, sex, underlying disease, laboratory data	NA		They measured vitamin D levels for multiple times, but we only focused on the initial baseline.
Park, Y. J., et al. (2017)	the Korean Health Technology R&D Project, Ministry of Health and Welfare, Republic of Korea (HI13C1232)	Historical cohort	This study investigated the impact of vitamin D deficiency on the development of infections after kidney transplantation.	We retrospectively investigated the medical records of 174 patients who had undergone kidney transplantation at Kyungpook National University Hospital between January 2011 and December 2013. A total of 164 KTRs were	One hospital study / All KTRs were regularly examined at Kyungpook National University Hospital.	(ESRD) Patients who had undergone kidney transplantation were retrospectively included / no sampling	Patients underwent kidney transplantation; received immunosuppression treatment;	N=164 whose 25-hydroxyvitamin D (25(OH)D) levels were measured within 2 weeks prior to kidney transplantation.	Patients without vitamin D levels checked / didn't specify;	Serum vitamin D deficiency is defined as a 25(OH)D concentration < 50 nmol/L (20 ng/mL)	25(OH)D ? 50 nmol (20 ng/mL)	Primary	All infections, hospitalizations events, graft function, and mortalities were recorded.	The outcome maybe either clinical or laboratory based	A total of 164 KTRs were followed up for a mean of 24.8 ?? 10.7 months.	The mean concentrations of serum 25(OH)D in patients with and without vitamin D deficiency were 10.97 ?? 4.69 and 27.34 ?? 8.70 ng/mL, respectively	Deficiency: M94 (69.6) Not deficiency: M22(75.9)	Underlying disease: ESRD received renal replacement therapy	Deficiency: 16 Sufficiency: 2	NA	NI	Chi-square for CMV infection	NI	NI	No	included age, sex, body mass index (BMI), renal replacement therapy before kidney transplantation, etiologies of end-stage renal disease (ESRD), transplant type, do-nor age, cold ischemic time, and human leukocyte	NI	NI	No further information available for CMV infection; they used all bacteria infection in stead

Moscarelli, L., et al. (2016).	NI	Historical cohort	To evaluate the correlation of biopsy-proven acute rejection, CMV infection, BKV infection, with 1,25-Dihydroxyvitamin-D3 deficiency and the benefit of calcitriol supplementation before and during the transplantation.	May 2005 to August 2014	Single-center study / Only included patients treated at the Renal Unit, Careggi University Hospital, Florence, Italy	Retrospectively included patients followed at their Renal Unit / No sampling	Patients underwent kidney transplantation; all patients received induction therapy	Patients underwent kidney transplantation at the hospital,	Patients treated with calcimimetics or vitamin D analogs, patients with early graft loss, patients with primary non-function, or patients switched to other therapies	Vitamin D supplementation: patients with oral calcitriol for at least one month before transplantation, and were prescribed calcitriol after transplantation	Patients who were not prescribed vitamin D in the last months before transplantation and during follow-up, patients who didn't assume calcitriol, and patients were not prescribed calcitriol	Primary	Laboratory-based CMV infection: CMV DNA in blood was detected using PCR. CMV infection was defined as a blood viral load > 100000 copies/mL or a 10 times increment over one-week interval.	From 2005 May, universal CMV prophylaxis is with acyclovir was replaced by three months' valgancicl ovir given only to donor-positive/recipient-negative cases or those treated with ATG (Thymoglobulins)	N=360, 1 year	mean recipient age at treatment: 51 (23-70)	M: n=252, 70%; F: n=108, 30%	All with primitive renal disease, such as glomerulonephritis, interstitial nephritis, DM, ADPKD, various other specified and unspecified	User group: n=4, 3% Control group: n=21, 9%	Mean viral load in user: 3.5+-1.5*10^4 Mean viral load in control: 13.7+-4.1*10^4	NI	Fisher's exact test for categorical variables, Cox proportional hazards regression to identify independent risk factors	No calcitriol exposure: HR=1.91, CI=1.17-3.13	No calcitriol exposure: HR=2.31, CI=1.44-3.71	No	serum 1,25(OH)2 D3 deficiency ,BPAR(biopsy-proven acute rejection), BKV infection, CMV serostatus, Steroid boluses, peak PRA>10%, mean cold ischemia time, BMI>25, recipient gender, donor gender, donor-recipient	serum 1,25(OH)2 D3 deficiency ,BPAR(biopsy-proven acute rejection), BKV infection, CMV serostatus, Steroid boluses, BMI	They also compare the CMV infection and viral load at 1, 6, 12 months after transplantation (Table 5). The vitamin D level was always higher among user group, and the CMV viral load was lower in user group at month 1 and 6.
Lee, J. R., et al. (2014)	National Center for Advancing Translational Sciences, National Institutes of Health, through the Weill Cornell Clinical and Translational Science Center, K08-DK087824 (T.M.) from the National Institutes of Diabetic and	Historical cohort	To investigate the association between post-transplant vitamin D status and the incidence of acute cellular rejection, CMV disease, and BK virus nephropathy during the first year of transplantation and kidney allograft function at 1 year in	January 2005 to December 2010; each patient were followed for 1 year	Single-center study/only patients who received a kidney transplantation at the New York Presbyterian Hospital ?V Weill Cornell Medical Center during the period of January 2005 to December 2010 were included	1211 patients who received kidney transplantation at the New York Presbyterian Hospital ?V Weill Cornell Medical Center during the period of January 2005 to December 2010 were included; no sampling	Patients received kidney transplantation;	Only 351 patients who had circulating levels of 25(OH)D measured within the first 30 days of transplantation and performed a retrospective cohort study to assess the impact of vitamin D status on renal allograft outcomes.	Patients without serum vitamin D level checked within 30 days of transplantation	Serum vitamin D deficiency: 25(OH)D levels <= 20 ng/mL (50 nmol/L)	Serum Vitamin D sufficiency: 25(OH)D levels > 20 ng/mL (50 nmol/L)	Secondary	Laboratory-based: CMV disease diagnosis was based on a positive blood PCR or pp65 antigenemia and evidence of organ involvement.	They also recorded 1,25(OH)2 D3 vitamin D treatment: 78 patients received vitamin D supplementation: taking ergocalciferol or cholecalciferol initiated by the transplant physician within the first 90 days of transplantation.	N=351; followed for 1 year	Mean age: 52.3 (+-13.6)	F: 130 (37%) M: 221 (63%)	All patients received kidney transplantation; 95(27.1%) patients had DM, 38 (10.8%) patient received prior kidney transplantation,	Deficient group: N=13 (6%) Sufficient group: N=8 (5.9%) P=0.99	NA	NI (historical cohort)	Fisher's exact test for CMV disease	NA	NA	No	1,25 vitamin D treatment, age, sex, African American race, deceased donor transplantation, preemptive treatmen	Not for CMV infection	They further stratified by CMV donor/recipient antibody status, and there is no significant difference in CMB incidence between the vitamin D deficient group and the vitamin D sufficient group (Table S1)

Fernandez-Ruiz, M., et al. (2019).	This research was supported by ??Plan Nacional de I+D+I?? and Instituto de Salud Carlos III (Proyecto Integrado de Excelencia [PIE] 13/00045), Subdirecci?n General de Redes y Centros de Investigaci?n	Prospective cohort	To attempt to replicate the findings by Astor et al, we have reanalyzed our database by taking into account any episode of late (>6 mo after transplantation) CMV infection regardless of the presence of symptoms or the need of antiviral therapy.	November 2014 to December 2016; followed at least 12 months	Single-hospital based study; included patient at Hospital Universitario ??12 de Octubre?? (Madrid, Spain).	Patients underwent kidney transplantation during November 2014 to December 2016	Adults received kidney transplantation, aged 53+-15.4	Consecutive adult patients (18 years and older) with ESRD undergoing KT during this period and providing informed consent were deemed eligible for inclusion. Only patients with 25(OH)D measurement at post-transplant month 6 were included (n=215).	Double organ recipients were excluded. Graft loss and death cases were also excluded.	Serum vitamin D levels: vitamin D deficiency 25(OH)D levels <20 ng/mL (50 nmol/L)	Serum Vitamin D levels > 20ng/mL	Primary	The diagnosis of cytomegalovirus (CMV) disease required the demonstration of viral replication and the presence of attributable symptoms, and was further categorized as viral syndrome or end?organ disease.	According the corresponding author, the setting of this letter is the same as their previous article Fernandez-Ruiz, M., et al. (2018) "Vitamin D deficiency and infection risk in kidney transplant recipients: A	N=215, followed-up for at least 12 months	54.3+-15.4 in previous study; NI for 215 patients	M=171 in previous study; NI for 215 patients	ESRD; Some pre-transplant chronic comorbidities such as DM, COPD...	Vitamin D deficiency: 34/135, 25.2% without deficiency: 14/80, 17.5% P=0.191	NA	NI	Categorical variables were compared using the Chi-square test	NA	NA	No	Age, nutritional status, previous KT, glomerulonephritis as ESRD, duration of pre-transplant dialysis, use of t-cell depleting agent	NI	They further exclude high risk patients (D+/R-), and they found that the incidence was higher of late CMV infection in recipients with vitamin D deficiency	Use the information from the previous study
Chao, C. T., et al. (2012)	None declared.	Case-control study	Assessing the risk factors of herpes zoster reactivation in hemodialysis patients	1 January, 2000 to 31 December, 2009	Single-centre study, recruited patient from their dialysis unit	All patients received dialysis in their unit, either they're in-patient or outpatient. These patients were identified by ICD code and chart-review.	Patients receiving H/D; older than 20 years	All dialysis patients, over aged 20 years, visit the hospital, either in-patient or outpatient between 01/Jan/2000 to 31/Dec/2009 were identi?ed by ICD-9-CM (International Classi?cation of Diseases, Ninth edition, Clinical Modi?cation) diagnostic codes of 585.x (chronic renal failure), 403.x (hypertensive renal disease), 404.x (hypertensive renal and heart disease) and V45.11 (renal	Those who received dialysis due to acute renal failure, who were dialyzed for less than 3 months, who had history of solid organ transplantation, and those with concomitant pregnancy were excluded.	Vitamin D supplementation: Use of 1?\-hydroxylated vitamin D --- Concomitant medication, either through oral, intravenous or subcutaneous route, was de?ned as the use of such medication starting at least 1 month before the index event. Drug compliance was established with thrice weekly dialysis clinic visits and pill counts.	No vitamin D supplementation	Primary	Clinical-based diagnosed herpes zoster: From this cohort, we further recruited patients with herpes zoster reactivation, through cross-matching with ICD-9-CM diagnostic code of 053.x (herpes zoster).Subsequent chart		Total N=126 (Case: N=63; Control: N=63); followed from 01/Jan/2000 to 31/Dec/2009	Case (with HZ): 62 (27.0-85.0); Control (without HZ): 63 (29.0-88.0)	M: 44 (35%) F:82 (65%)	All had renal diseases caused by DM, HTN, glomerulonephritis, ot other causes	Exposure: 1?\-hydroxylated vitamin D use Exposure among case: N=3 (5.4%) Exposure among control: N=29 (46.0%)	NA	NA for case-control studies	multivariable-adjusted conditional logistic regression model for further analysis.	OR=0.08 (0.0?V0.3)	OR=0.06 (0.0?V0.4)	No	Demographic pro?les including age, sex, other concomitant comorbidities (diabetes mellitus [DM], hypertension, congestive heart failure [CHF], coronary artery disease [CAD], hepatic disease, any malignancy, and	Hepatitis or cirrhosis, Cerebrovascular accident, Use of iron therapy, Use of corticosteroids, use of statins, CRP, Intact PTH, Ferritin	A re-analysis excluding patients with SLE using corticosteroids or those with liver diseases did not in?uence our results signi?cantly.	A matched case-control study

Ban, T. H., et al. (2017).	Basic Science Research Program through the National Research Foundation of Korea (NRF) funded by the Ministry of Education, Science, and Technology (NRF-2013R1A1A1009718) and by a grant from the Korean Health	Historical cohort	To investigate whether pre-transplant 25(OH)D level could predict the incidence of acute rejection or infectious complications during the early post-transplant period.	January 2011 to December 2013	Single-centre study; 362 kidney transplants were performed in Seoul St. Mary's Hospital. In this study, we included patients with at least a 3-month follow-up duration at our center.	Retrospectively include patients receiving renal transplantation	Patients received renal transplantation	patients with at least a 3-month follow-up duration at Seoul St. Mary's Hospital.	Excluded patients who were immunologically high risk, such as those with a panel reactive antibody (PRA) value ??80%, positive results for either type of crossmatch test, presence of donor human leukocyte antigen (HLA)-speci?c antibodies (DSA), or ABO incompatible kidney transplantation with high baseline anti-	Serum 25(OH)D levels: the cutpoints were the tertile Middle tertile group (n = 57; 8.31?V12.1 ng/mL);mean 25(OH)D 10.3(1.2) ng/ml Low tertile group (n = 59; ??8.3 ng/mL); mean 25(OH)D 5.6(1.6) ng/ml; "rum 25(OH)D concentration was measured in the process of evaluating suitability for KT within 6 months of KT"//	High tertile group (n = 58; >12.1 ng/mL); mean 18.7 (11.4) ng/ml	Secondary	CMV was monitored at 1, 3, 6, 9, and 12 months after transplantation using a real-time polymerase chain reaction (PCR) method.	The cut-point of vitamin D levels are weird; even the high tertile is still low (30.25nmol/L) All patients received immune suppression treatment	N=174 were recruited; median follow-up period 35.5 (8.3) months	mean age ranged from 42.7-73.4 Low tertile group: 42.8+-10.7 Middle tertile group: 42.7+-13.8 High tertile group: 43.4+-12.0	Low tertile group: M:28(47.5%) F: 31 Middle tertile group: M35 (61.4%) F:22 High tertile group: M42 (72.4%) F:16	Kidney transplantation and immunosuppression therapy	CMV case numbers: Low: 3 (5.1%) Middle: 9 (15.8%) High: 9 (15.8%) Herpes zoster: (?????) Low: 6 (10.2%) Middle: 3 (5.3%) High: 6 (10.3%)	NA	NI	Fisher's exact test	NA	NA	No	Sex, age, blood type, HLA, DM, HTN, osteoporosis, iPTH, Hb, Cr,CA,P,TC,TG,LDL	NA	NI for infection	Their definition of vitamin D levels were different from the normal value
Astor, B. C., et al. (2019)	The authors declare no funding or conflicts of interest.	Historical cohort	whether 25(OH)D level is associated with CMV infection in kidney transplant recipient.	01/Jan/2004-30/Jun/2014;	Single-centre cohort	The Wisconsin Allograft Recipient Database was initiated in 1984 to collect information on all solid organ transplants performed at the University of Wisconsin. They only included patients received kidney transplantation	patients received kidney transplantation, older than 18 years	All patients who received a primary kidney transplant at the University of Wisconsin between January 1, 2004, and June 30, 2014, were at least 18 years of age at the time of transplantation and survived with a functioning graft for at least 6 months after transplantation were eligible for inclusion in this study.	Patients younger than 18 years and didn't survive with a functioning graft for at least 6 months	Serum 25(OH)D were checked least 6 months after transplantation. All 25(OH)D measurements available after this time were used. Vitamin D insufficiency: 25(OH)D 20?V29 vitamin D deficiency: 25(OH)D <20 g/mL	Serum 25(OH)D ?30 ng/mL	Primary	Laboratory-based criteria: CMV quantitation was done using a real-time PCR procedure; defined as any detected value of plasma CMV above the laboratory reference (250 copies/mL). (CMV PCR is monitored if there	Recipients with CMV infection before the first 25(OH)D measurement were excluded. CMV prophylax is at our institution is with valganciclovir for 6 months after transplantation, and acyclovir was used in low-risk patients	1976 recipients survived with 25(OH)D measurement between 2004-2014; (recipients were followed for up to 1 year after vitamin D checked or 31 December 2015)	Overall average age: 50 years old	Female: 41.1% Male: 58.9%	ESRD caused by DM, HTN, PKD, glomerulonephritis	NI; A total of 107 recipients developed CMV during follow-up; 76 of them received CMV treatment	NA	NI	Multivariate Cox proportional hazard regression models	Serum 25(OH)D ?30 ng/mL: reference Serum 25(OH)D 20-29 ng/mL: 1.07 (0.69-1.69) Serum 25(OH)D <20 ng/mL: 2.02 (1.22-3.33)	Serum 25(OH)D ?30 ng/mL: reference Serum 25(OH)D 20-29 ng/mL: 1.10 (0.74-1.70) Serum 25(OH)D <20 ng/mL: 1.81 (1.06-3.09)	Yes	Age, sex, race, cause of ESKD, BMI, donor status, prior transplant, delayed graft function, induction immunosuppression, smoking status, HLA mismatch category, CMV serostatus, time from transplant to 25(OH)D	Adjusted for age, sex, race, cause of ESKD, BMI, donor status, prior transplant, delayed graft function, induction immunosuppression, smoking status, HLA mismatch category, CMV serostatus, time from transplant to	1.Only considering treated CMV events: The association of 25(OH)D deficiency with incidence of CMV was similar in analyses considering only treated CMV events (n = 76 events; adjusted RH = 1.73; 95% CI, 1.00-	RH: adjusted relative hazard The exposure time was at least 6 months after transplantation, which was different from other studies.
